# Intraannular photoreactions in *pseudo*-geminally substituted [2.2]paracyclophanes

**DOI:** 10.3762/bjoc.7.78

**Published:** 2011-05-24

**Authors:** Henning Hopf, Vitaly Raev, Peter G Jones

**Affiliations:** 1 Institut für Organische Chemie, Technische Universität Braunschweig, Hagenring 30, D-38106 Braunschweig, Germany, Fax: +49 531 / 391 5388; 2 Institut für Anorganische und Analytische Chemie, Technische Universität Braunschweig, Hagenring 30, D-38106 Braunschweig, Germany, Fax: +49 531 / 391 5387

**Keywords:** paracyclophanes, photoadditions, photoisomerizations, proximity effects, topochemical reaction control, vinylcyclopropanes, X-ray structural analysis

## Abstract

The photoisomerization of the pseudo-*geminal* tetraene **11** furnishes the cyclooctadiene derivatives **13** and **15** with a completely new type of molecular bridge for a [2.2]paracyclophane which promise many interesting novel applications; the same is true for the photoisomerization of **22** to **23** and **24**. The structures of these new hydrocarbons were established by X-ray crystallography and spectroscopic analysis; among the noteworthy structural features of **13** and **15** are unusually long carbon–carbon single bonds (>1.64 Å).

## Introduction

Photodimerizations of crystalline aromatic or olefinic compounds are among the oldest known organic photoreactions. In this type of reaction the crystal lattice locks the relative orientation of the substrate molecules or their photoreactive groups. If the orientation is favorable for reaction, reactivity increases. Unlike photochemistry in homogeneous solution, this often leads to highly selective formation of the photoproducts. Schmidt coined the term “topochemical principle” or “topochemistry” for (non)reactivity determined by a limiting distance between the reactive groups [2–4]. Although the model found widespread acceptance, many exceptions to the concept were known from the very beginning [5]. Later, AFM techniques enabled experimental elucidation of solid-state photochemistry. This showed that the supramolecular arrangement of molecules in the crystal plays a more important role for reaction control than the simple alignment of double bonds. Long-range molecular movements within crystals upon photochemical reaction and even topotactic single-crystal to single-crystal reactions were found, although the latter are rare. The subject has been comprehensively covered by recent reviews [6–8]. Reactions of inclusion complexes are a variation of the solid-state photochemistry topic [9]. Here, co-crystals of a host compound and the starting materials of a photochemical reaction are used and the supramolecular arrangement [10] may control the regio- and stereoselectivity of the photo-process. The enantioselective photochemical conversion of chiral crystals into optically active products has also been described [11]. Some approaches utilize zeolites as supramolecular hosts for photoreactions [12–14]. Internal complexation, or intracrystalline adsorption, occurs by diffusion of the guest into the channels and cavities of the zeolite crystal and is size- and shape-selective. Complexation of organic compounds may reversibly depend on temperature. The geometry of zeolite cavities restricts conformation and orientation of included guests and their reaction partners and leads to more selective reactions. In the absence of any low-energy electronic states of the zeolite, photoreaction occurs only with the included guest.

The common disadvantage of solid-state photoreactions is the difficulty in predicting and controlling reaction selectivity. It remains a challenge to find the suitable crystal, co-crystal, or inclusion complex for the desired regio- or stereoselective outcome of a given reaction. Therefore, an attractive strategy is to transfer the topochemical control from the solid state to a homogeneous solution using suitable templates. Such reactions are easier to analyze, design, and optimize.

Templated photochemistry in solution is possible if the photoreactive moieties can be brought into suitable positions for reaction. Such an arrangement may in principle be reached either by non-covalent bonding (e.g., hydrogen bonds) or by (cleavable) covalent bonds. The latter case can be realized if two (or more) reactive moieties are attached to a rigid scaffold, which is able to fix them in the correct position for reaction.

**Scheme 1:**
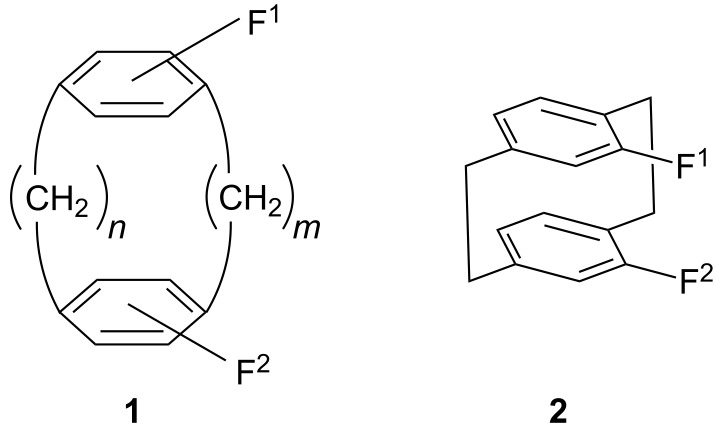
[2.2]Paracyclophanes as scaffolds for intraannular photodimerization reactions in solution.

One such system is the generalized paracyclophane molecule **1** shown in Scheme 1. Here the distance between the benzene “decks” carrying the functional groups F^1^ and F^2^ can be adjusted both by the length of the two molecular bridges (variation of *m* and *n*), and by the relative orientation between these groups in terms of their relative positions in the aromatic subsystems. Although there will never be a continuum of intrafunctional distances, numerous spatial arrangements of F^1^ and F^2^ are possible, keeping in mind that, for example, the molecular bridges of **1** – with the number of carbon atoms held constant – can be modified by introducing functionality into this part of the molecule, making the bridges more rigid, and/or by exchanging the benzene rings of **1** for other aromatic or heteroaromatic subsystems. The two bridges do not have to be of the same length nor the aromatic nuclei of the same type.

**Scheme 2:**
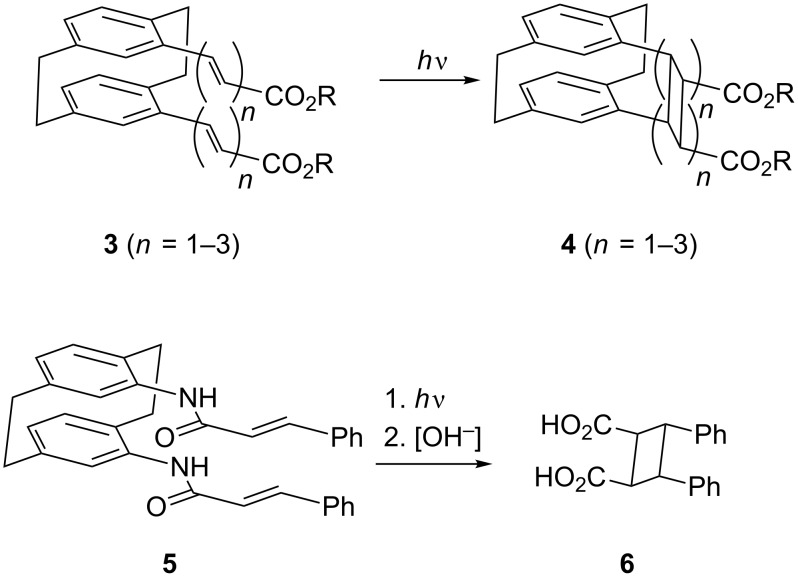
Stereospecific intramolecular [2+2]photoadditions using [2.2]paracyclophane spacers.

In our work we have so far concentrated our efforts on derivatives of [2.2]paracyclophane (**1**, *m* = *n* = 2) with the two functional groups usually in the so-called *pseudo*-geminal positions, that is, directly above each other as shown in **2**. The intraannular distance is approximately 3.1 Å in [2.2]paracyclophane and hence is less than the separation of the layers in graphite (3.4 Å) or between the base pairs of DNA (3.34 Å) [15]. In other words, the distance between the benzene rings of [2.2]paracyclophane and consequently of the two functional groups directly bonded to them is just slightly shorter than the length of a p-orbital, an ideal prerequisite for an intraannular reaction to take place should other factors, such as excessive strain, not prevent it. In principle, cyclophanes such as **1** are thus excellent model compounds for “molecular workbenches” [16–19] and we have already shown that certain *pseudo*-geminally substituted derivatives can be used as proxies for the crystal lattice in various solid-state reactions [20–22]. For example, on irradiation the unsaturated esters **3** photocyclize in excellent (up to quantitative) yield to the ladderane derivatives **4**. In this case the cyclophane moiety is the “order-generating” part of the molecule and the originally flexible, unsaturated chain remain attached to each other by stable C–C-bonds; altogether the process amounts to a stiffening (rigidization) of the molecules **3**. In the case of the bis amide **5** (Scheme 2), photodimerization to the corresponding cyclobutane derivative occurs readily, and the photoproduct can be saponified to the corresponding *pseudo*-geminal diamine and truxinic acid (**6**) in excellent yield, thus allowing its stereospecific synthesis. We believe that the use of the [2.2]paracyclophane scaffold as a removable spacer can be developed considerably further for the stereospecific synthesis of many other compounds.

## Results and Discussion

**Scheme 3:**
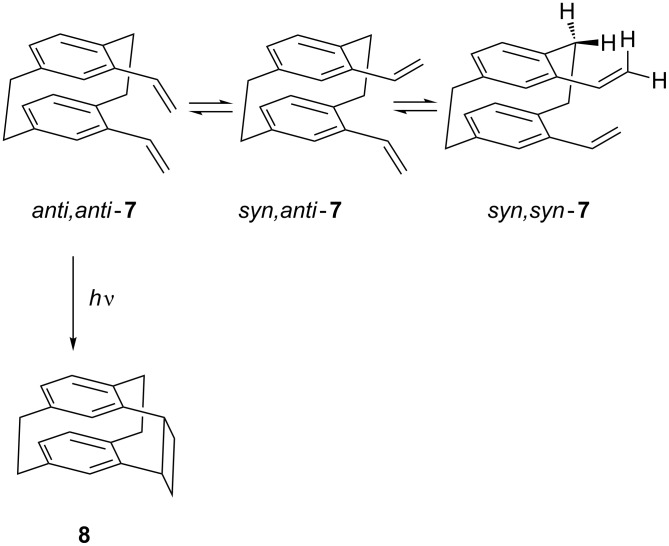
Different conformations of *pseudo*-geminal divinyl[2.2]paracyclophane.

However, the detailed stereochemical situation is in fact more complex, and the origin of the stereospecificity requires a more thorough analysis. For example, we have shown [22] by time-resolved photoelectron spectroscopy (TR-PES) that the *pseudo*-geminal divinyl derivative **7** can only react from its *anti*,*anti*-conformation (*anti* referring to the orientation of the vinyl substituent to the neighboring ethano bridge) to yield the cyclobutane derivative **8**. The *syn*,*anti*-conformation, which has been shown to be present as a conformer in the solid state by X-ray structural analysis does not photocyclize to **8**. Moreover, *syn*,*syn*-**7** is evidently too sterically hindered (by repulsion of the relevant hydrogen atoms as shown in Scheme 3) to be part of the conformational equilibrium.

**Scheme 4:**
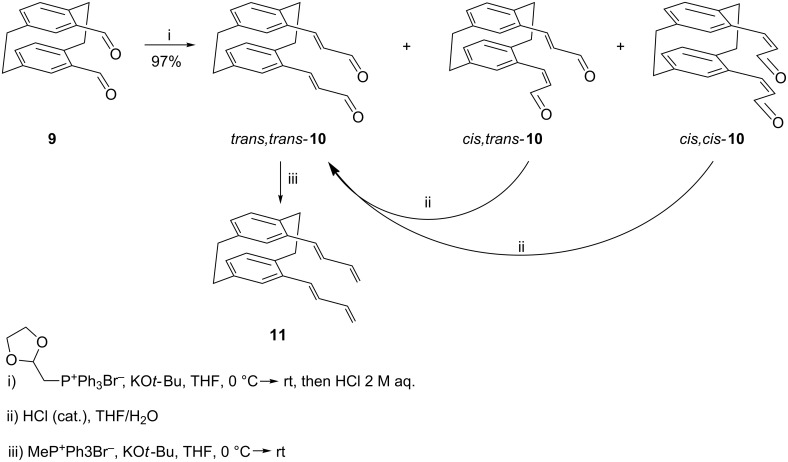
Preparation of tetraene **11**.

Clearly, the situation is conformationally much more complex in cases such as the triene esters **3**, where several conformations could be present in the ground state. To investigate this phenomenon we decided to simplify our substrates structurally and chemically by omitting any functional groups. In this contribution we report on the results obtained with two hydrocarbons **11** (Scheme 4) and **16**.

Bis-ene-al **10** was obtained in excellent yield (97%) as a mixture of three isomers (Scheme 4) in a ratio of 70:15:1; the isomers were isolated by column chromatography and their structures were established from their spectroscopic data, especially from their NMR spectra (see Experimental). Treatment of the *cis,trans*- and *cis,cis*-isomers of **10** with hydrochloric acid in aqueous THF converted them into the thermodynamically most favorable *trans,trans*-isomer.

Bis-diene **11**, which was obtained in virtually quantitative yield from *trans,trans*-**10** by a Wittig olefination, appears to be unstable in the solid state at room temperature, but in the refrigerator at −20 °C or in dilute (~0.1 M) solution in dichloromethane or chloroform it can be stored in the dark for at least 3 months without any detectable decomposition or polymerization.

**Scheme 5:**
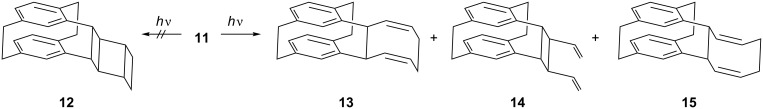
Photolysis of tetraene **11**.

**Figure 1:**
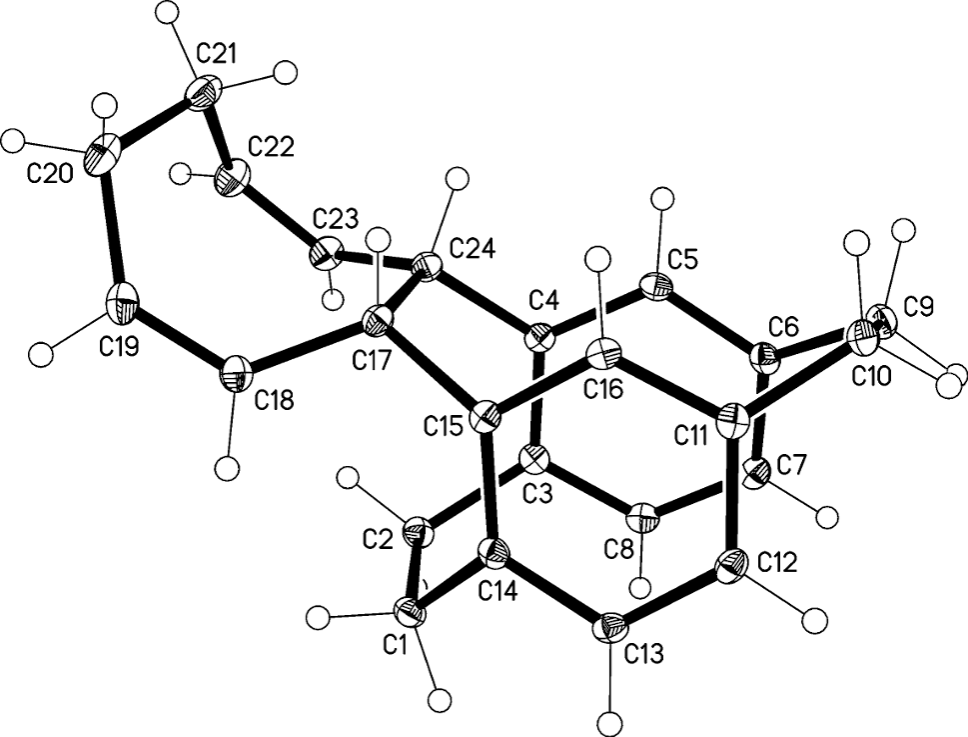
The molecule of compound **13** in the crystal. Ellipsoids correspond to 30% probability levels.

Irradiation of **11** with a halogen lamp (1 kW, 10 cm distance, water cooling) for 16 h gave a mixture of products (Scheme 5), which contained two isomers of a cycloocta-1,5-diene derivative, **13** (as the main product) and **15** (*syn-* and *anti-* position, respectively, relatively to the bridge) together with the divinylcyclobutane derivative **14** in moderate yield (total yield 70%, ratio **13**:**14**:**15** = 43:5:8 by ^1^H NMR analysis). The expected ladderane **12** was not detected in the reaction mixture by NMR spectroscopy. Separation by column chromatography gave the pure divinylcyclobutane derivative **14**, but the cyclooctadienes were not separated from each other. Fractional crystallization of the mixture of cyclooctadienes from CHCl_3_/MeOH mixture gave an analytically pure sample of **13**, which was characterized by single-crystal X-ray diffraction (Figure 1).

Further irradiation of compounds **13**, **14** and **15** did not lead to any detectable photoproducts.

**Scheme 6:**
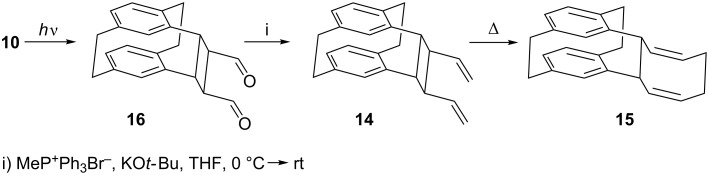
Photolysis of *trans*,*trans*-dienal **10**.

The success in preparing cyclobutane derivative **4** (*n* = 1) from the corresponding cinnamophane diester **3** (*n* = 1, quantitative yield) led us to attempt to prepare the corresponding cyclobutane dialdehyde derivative **16** (Scheme 6). Unfortunately, although this was the only product after 2 h of irradiation with a halogen 1 kW lamp, it appeared to be very unstable even below 0 °C, although it was stable enough for NMR identification. Wittig olefination of the irradiated mixture gave the divinylcyclobutane derivative **14** as the sole product and was isolable by column chromatography.

Interestingly, all three isomers of **10** (*trans,trans*-, *cis,trans*- and *cis,cis*-) under the above irradiation conditions furnish the same product: **16**. It is hence likely that a rapid photoequilibration process precedes the ring closure to the final product.

**Figure 2:**
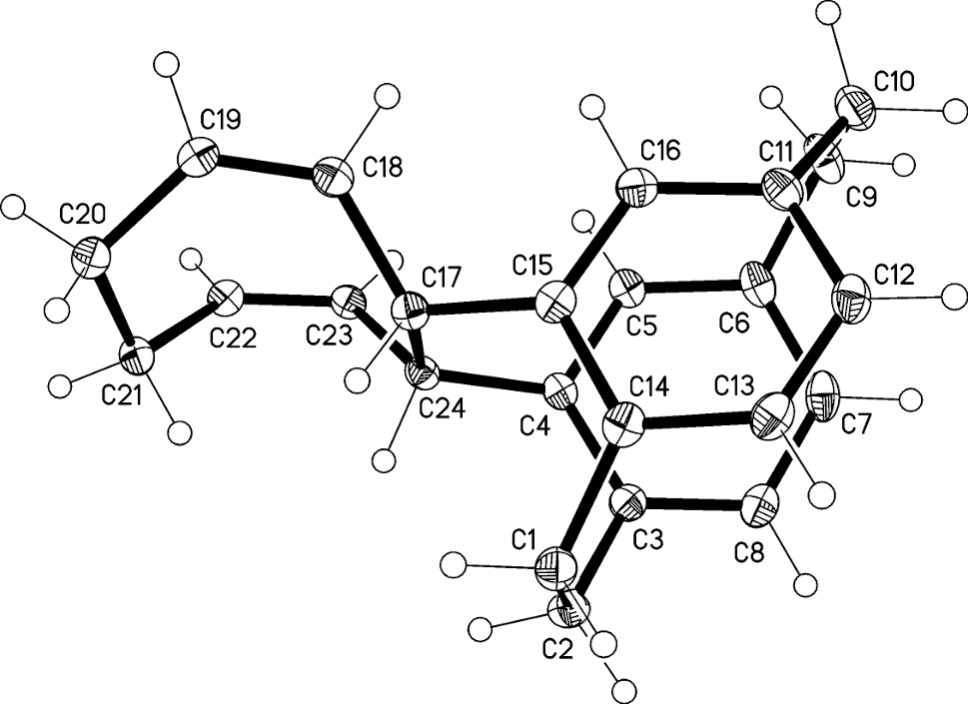
The molecule of compound **15** in the crystal. Ellipsoids correspond to 30% probability levels.

Attempts to crystallize **14** from boiling ethanol led to a mixture of **14** and the cyclooctadiene derivative **15**, which was separable by column chromatography. The divinylcyclobutane derivative **14** was completely converted into the cyclooctadiene derivative **15** within half an hour in boiling ethanol. The structure of **15** was confirmed by single-crystal X-ray analysis (Figure 2).

Molecules of **13** and **15** show common structural features. Despite the introduction of the new bridge C17–C24, the form of the original [2.2]paracyclophane is maintained to a considerable extent, with a flattened boat conformation of both six-membered rings (C4,5,7,8 and C12,13,15,16 remain essentially coplanar). However, the rings become significantly non-parallel (interplanar angles 14.4 and 13.4°, respectively). The new bridges C17–C24 are extremely long at 1.643(2) and 1.652(2) Å, respectively, even longer than the previously present bridges C1–C2 and C9–C10 at 1.57–1.60 Å. The steric crowding of **13** associated with the *syn* geometry is shown by, e.g., the short contact H18^…^H1A 1.91 Å; compound **15** has no intramolecular H^…^H < 2 Å.

**Scheme 7:**
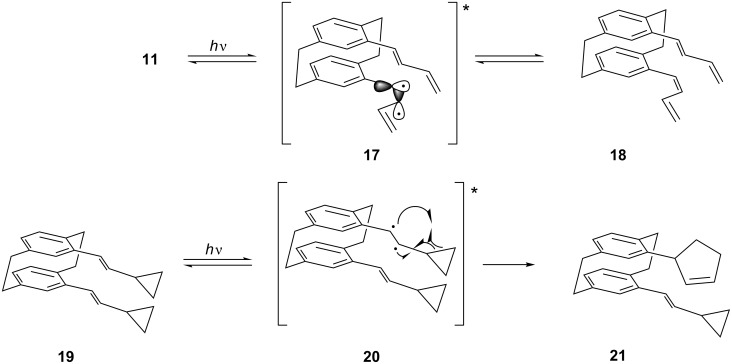
*Cis*–*trans*-isomerizations of the double bonds of the *pseudo*-geminal cyclophanes **11** and **19**.

Form the stereochemical viewpoint the above photocyclizations are quite complex. Not only can the *pseudo*-geminal substituents in principle adopt different conformations in the ground state, because of possible rotation around the various σ-bonds, but this situation becomes even more intricate when the substrates are photochemically excited. For example, on photoexcitation diradicals **17** (Scheme 7) should be the intermediates in conceivable *cis*–*trans-*isomerizations, e.g., **11**→**18**, and these diradicals could undergo very different subsequent reactions (in which, of course, it could also be of importance whether these intermediates are singlets or triplets).

To test for the possible formation of radical intermediates in the above photocyclizations, we decided to prepare the biscyclopropane analog of **11**, the bisvinylcyclopropane **19** (or one of its *cis*-isomers) and subject this presumably strained hydrocarbon to our photocyclization conditions. Of course, this system also has various options to react, among them the photoisomerization to a mono- or all-*cis*-diastereomer. If this process took place, it would involve the diradical **20**, which could isomerize to **21** with release of strain. The process could also occur a second time to provide a *pseudo*-geminally substituted [2.2]paracyclophane, now carrying two cyclopentenyl substituents. Should these ring-enlarged paracyclophanes not be observed, this would not necessarily constitute a proof against diradical(oid) intermediates in these reactions. However, if derivatives such as **21** were among the photoproducts the involvement of radicals in the photoisomerizations would be indicated.

**Scheme 8:**
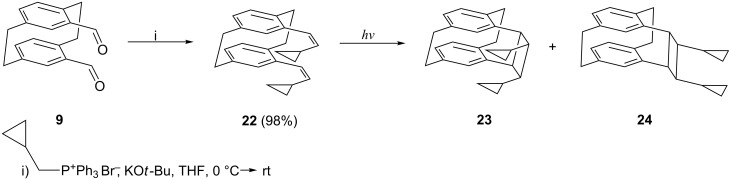
Preparation of the vinylcyclopropanes **22**–**24**.

**Figure 3:**
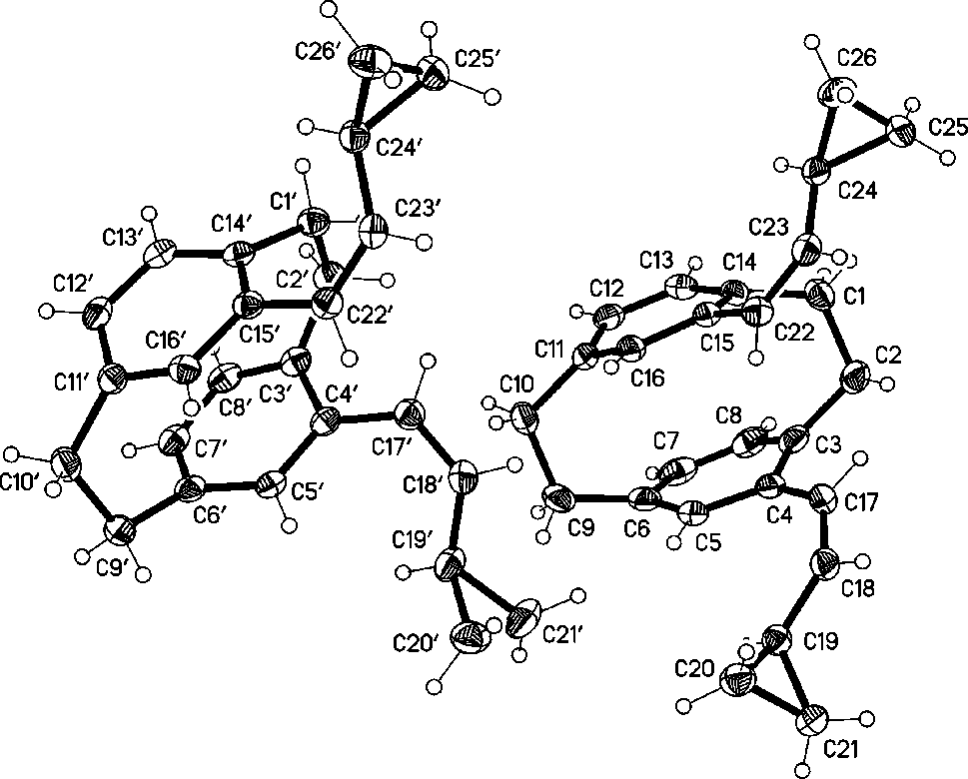
The two independent molecules of compound *Z*,*Z*-**22** in the crystal. Ellipsoids correspond to 50% probability levels.

We therefore reacted the bis-aldehyde **9** with the ylide prepared from cyclopropylcarbinyl triphenylphosphonium bromide and obtained in quantitative yield a product mixture consisting of the three possible diastereomers *E*,*E*-, *E,Z*- and *Z,Z*-**22** (Scheme 8), the latter being the main product as is often observed in classical Wittig reactions (product ratio 1:13:31; analysis by ^1^H NMR spectroscopy, see Experimental). The main product was separated by silica gel chromatography and its structure determined by X-ray crystallography (Figure 3).

The two independent molecules in the asymmetric unit are similar, with an r.m.s. deviation of 0.3 Å for all non-H atoms. As would be expected, the substituents are directed outwards from the ring systems. The non-bonded distances C17^…^C22 and C18^…^C23, across which bonds are to be formed are 3.34, 3.35 and 5.12, 4.93 Å; clearly the latter, in particular, can be reduced by suitable rotations.

**Figure 4:**
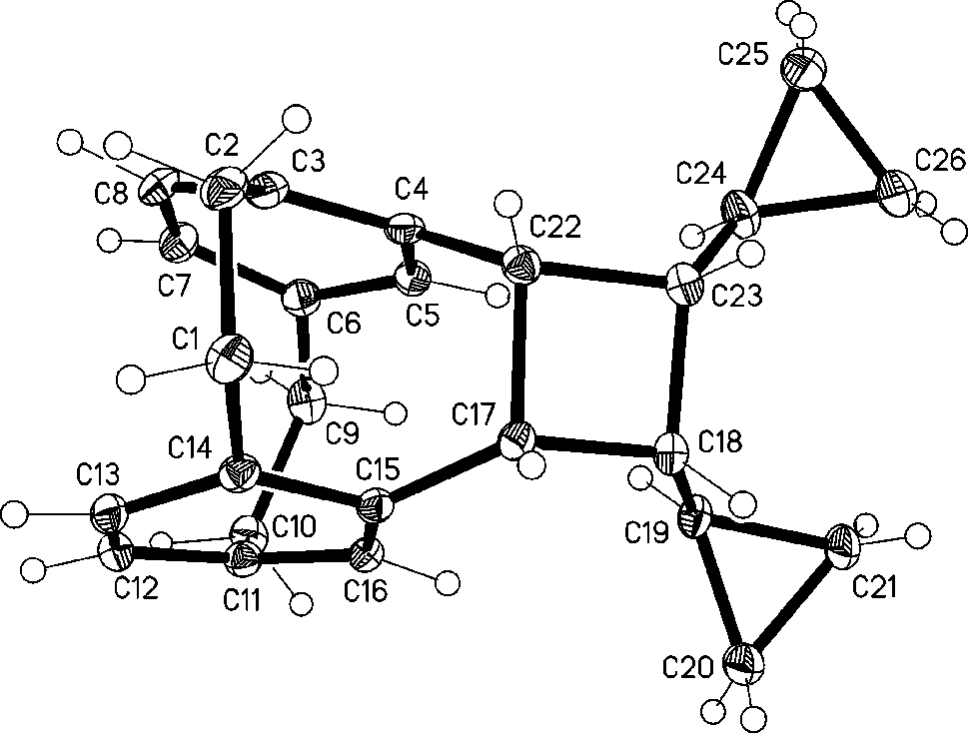
The molecule of compound **23** in the crystal. Ellipsoids correspond to 50% probability levels.

**Figure 5:**
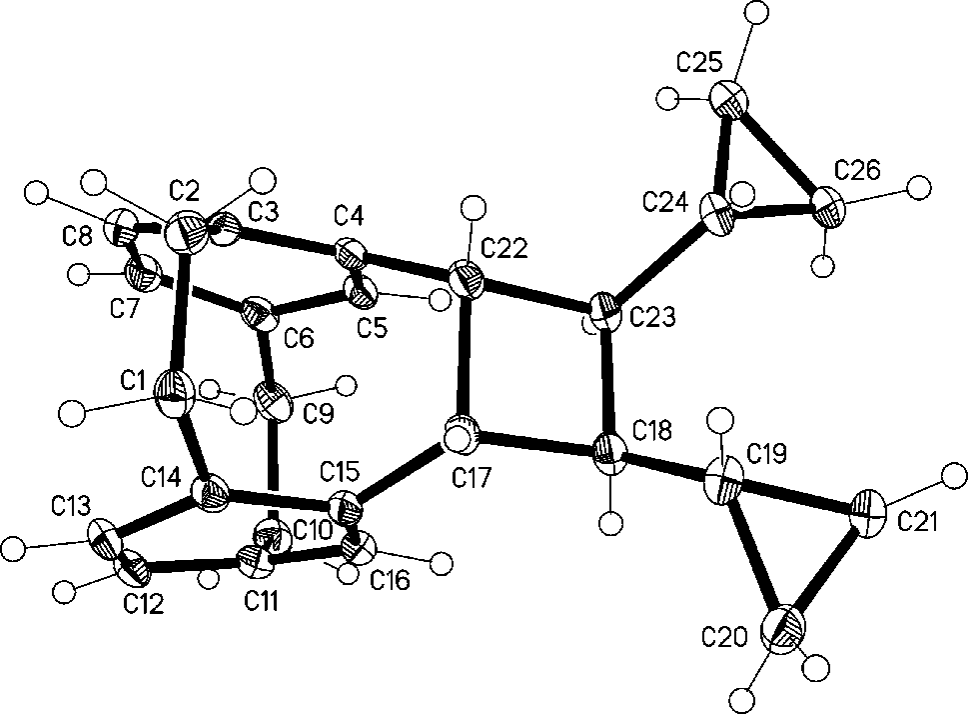
The molecule of compound **24** in the crystal. Ellipsoids correspond to 30% probability levels.

Irradiation of *Z*,*Z*-**22** with a 1 kW halogen lamp in a Pyrex flask over 12 h (Scheme 8) gave only two [2 + 2] cycloaddition products: The hydrocarbons **23** and **24** in 3:5-ratio with a total yield of 70%. The isomers were separated by column chromatography and their structures established by NMR spectroscopy and single-crystal X-ray analysis (Figure 4 and Figure 5); no other products could be detected.

As for molecules **13** and **15**, but to a slightly lesser extent, the newly formed bridges C17–22 in **23** and **24** are significantly longer than a standard single bond at 1.612(2) and 1.614(2) Å, respectively. On the other side of the four-membered rings, the bond lengths C18–23 relax to 1.563(2) and 1.559(2) Å. The interplanar angles between the six-membered rings of the original [2.2]paracyclophane unit are 12.9 and 12.7°.

These results clearly show that the photocylization occurs from the conformation in which the two *pseudo*-geminal substituents are rotated away from the nearest ethano bridge (*anti*,*anti*-conformation). The conformation with both of these groups *syn*-oriented towards this bridge, although in principle possible, is evidently not populated. Although in the crystalline state a *syn*,*anti*-conformation is preferred (Figure 3), no reaction takes place from this orientation on irradiation in solution. Since we have already demonstrated that a comparable situation prevails for the simplest compound studied in this series, hydrocarbon **7** (Scheme 3), we conclude that reaction from this *anti*,*anti*-conformation is the generally preferred reaction mode for derivatives of type **3** (Scheme 2). The production of **24**, however, proves that the stereochemical information contained in the first double bond (*E* or *Z*) can be lost in the course of the photochemical reaction. Whereas this *Z*→*E-*isomerization process must involve a diradical intermediate of type **17**, its lifetime is evidently too short to allow ring-expansion as depicted in Scheme 7. Whether this process might be induced thermally (vinylcyclopropane→cyclopentene rearrangement; [23]) is an open question.

## Conclusion

Although the detailed mechanisms of the photoisomerization of the tetraene **11** to the cyclooctadiene-bridged cyclophanes **13** and **15** and the iosmerization of **22** to **23** and **24** remain to be established, these processes allow the introduction of a completely new type of additional bridge into [2.2]paracyclophanes. For several of these new polycyclic molecules interesting preparative applications are conceivable, and we hope to report about them in the not too distant future.

## Experimental

*General*: Melting points: Büchi 530 melting point apparatus, uncorrected. Thin layer chromatography (TLC): Macherey–Nagel Polygram SilG/UV254. Column chromatography: Merck Kieselgel 60 (70–230 mesh). IR: Perkin–Elmer 1420 or Nicolet 320 FT–IR spectrometer. ^1^H and ^13^C NMR: Bruker AC 200 (^1^H) and 50.3 MHz (^13^C) in CDCl_3_, internal standards: TMS, δ = 0 ppm for ^1^H, CHCl_3_, δ = 77.05 ppm for ^13^C spectroscopy. UV–vis: Beckman UV 5230 or Hitachi U 3300. The samples were degassed by the freeze, pump, and thaw technique. Irradiations were conducted with a high-pressure mercury lamp (150 W) or a halogen torch lamp (1 kW) using water cooling reactor.

*Synthesis*: (1,3-Dioxolan-2-ylmethyl)triphenylphosphonium bromide was prepared according to [24]; 4,15-diformyl[2.2]paracyclophane (**9**) was prepared according to [20] with a modified oxidation step (Swern oxidation rather than the Dess–Martin protocol); 4,15-bis[(*E*)-2-formylvinyl]-[2.2]paracyclophane (*trans,trans*-**10**) was prepared according to [20]; cyclopropylmethyltriphenylphosphonium bromide was purchased from ABCR; methyltriphenylphosphonium bromide was purchased from Acros. Reagents were used without further purification. Solvents used were of analytical grade; anhydrous THF was distilled from an LiAlH_4_ dispersion with triphenylmethane as indicator.

**4,15-Divinyl[2.2]paracyclophane** (**7**): A freshly prepared solution of potassium *tert*-butoxide (4.26 g, 38.0 mmol) in anhydrous THF (50 mL) was added dropwise over 30 min to a cooled (ice/water bath), vigorously stirred dispersion of methyltriphenylphosphonium bromide (14.29 g, 40.0 mmol) in anhydrous THF (25 mL) under a N_2_ atmosphere. The bath was removed and the mixture stirred for 2 h at ambient temperature, then re-cooled to 0 °C, after which a solution of **9** (2.64 g, 10.0 mmol) in anhydrous THF (30 mL) was added dropwise over 1 h. The mixture was left to stir in the melting ice/water bath overnight and sat. aq. Na_2_SO_4_ solution (25 mL) added with vigorous stirring. The mixture was stirred for 15 min and the organic layer decanted. The aqueous layer was washed with THF (3 × 20 mL, decanting), then the combined organic phases were dried over anhydrous Na_2_SO_4_, filtered and concentrated under reduced pressure to give a solid residue (6.4 g). Column chromatography (50 mL of silica, CH_2_Cl_2_) gave 2.60 g (10 mmol, 100%) of pure hydrocarbon **7**. ^1^H NMR (200 MHz, CDCl_3_) δ 6.81 (dd, 2H, *J_1_* = 10.9, *J_2_* = 17.4 Hz), 6.60–6.40 (m, 6H), 5.36 (dd, 2H, *J*_1_ = 1.5, *J*_2_ = 17.4 Hz), 5.08 (dd, 2H, *J*_1_ = 1.5 , *J_2_* = 10.9 Hz), 3.60–3.40 (m, 2H), 3.05–2.86 (m, 6H) ppm; ^13^C NMR (50.3 MHz, CDCl_3_) δ 139.3, 138.0, 137.2, 135.5 (+), 134.6 (+), 132.4 (+), 129.8 (+), 114.6 (−), 35.0 (−), 32.5 (−) ppm; MS (EI, 70 eV) *m*/*z* (%): 261 (8), 260 (34), 131 (36), 130 (39), 129 (100), 128 (24), 115 (34).

**4,15-bis(butadien-1-yl)[2.2]paracyclophane** (**11**): A freshly prepared solution of potassium *tert*-butoxide (2.69 g, 24.0 mmol) in anhydrous THF (50 mL) was added dropwise over 30 min into the cooled (ice/water bath), vigorously stirred dispersion of methyltriphenylphosphonium bromide (8.57 g, 24.0 mmol) in 50 mL of anhydrous THF under a N_2_ gas flow. The bath was removed and the mixture was stirred for 1 h at ambient temperature, then re-cooled to 0 °C, after which a solution of **10** (0.95 g, 3.0 mmol) in anhydrous THF (30 mL) was added dropwise over 1 h. The bath was removed and the mixture was stirred for an additional 2 h. The resulting mixture was poured into a vigorously stirred mixture of ice (200 g), water (100 mL) and conc. (37%) aq. HCl solution (100 mL), and the mixture stirred until the ice had completely melted. The precipitate was suction filtered on a glass frit, washed with dilute (1:3) aq. HCl (3 × 30 mL) and water (3 × 30 mL), and dissolved in CH_2_Cl_2_ (100 mL). The organic solution was dried over Na_2_SO_4_, filtered and concentrated under reduced pressure without warming to give a colorless solid residue (0.94 g, 3.0 mmol, 100%) of hydrocarbon **11**, pure by NMR analysis. ^1^H NMR (200 MHz, CDCl_3_) δ 6.60–6.24 (m, 12H), 5.28–4.89 (m, 4H), 3.57–3.28 (m, 2H), 3.11–2.74 (m, 6H) ppm; ^13^C NMR (50.3 MHz, CDCl_3_) δ 138.7, 137.1 (+), 136.9, 136.7, 134.1 (+), 131.5, 131.1 (+), 129.7 (+), 129.2 (+), 116.1 (–), 34.4 (–), 32.0 (–) ppm.

Irradiation of 4,15-dibutadien-1-yl[2.2]paracyclophane – [2.2.2]tricyclophanes **13**, **14** and **15**: The solution of **11** (230.0 mg, 736 µmol) was irradiated by UV-lamp for 20 h. When the starting material was completely consumed (TLC monitoring), the reaction mixture was separated by column chromatography (silica, pentane) to give 14.3 mg of *bis*-vinyl derivative **14** and 145.8 mg of the mixture of cyclooctadienyl derivatives **13** and **15**. Total yield 160.1 mg (70%).

*Bis*-vinyl derivative **14**: ^1^H NMR (200 MHz, CDCl_3_) δ 6.48 (dd, 2H, *J*_1_ = 1.73, *J*_2_ = 7.79 Hz), 6.35 (d, 2H, *J* = 1.73 Hz), 6.23 (d, 2H, *J* = 7.79 Hz), 6.33–6.15 (m, 2H), 5.21–5.05 (m, 4H), 4.28–4.17 (m, 2H), 3.70–3.33 (m, 2H), 3.93–3.22 (m, 6H), 2.64–2.49 (m, 2H) ppm; ^13^C NMR (50.3 MHz, CDCl_3_) δ 140.1, 139.8, 139.5, 139.3 (+), 134.1 (+), 133.2 (+), 128.6 (+), 115.0 (−), 49.1 (+), 40.1 (+), 36.4 (−), 32.5 (−) ppm; MS (EI, 70 eV) *m*/*z* (%): 312 (8), 157 (31), 156 (41), 155 (100), 142 (12), 141 (56), 129 (16), 128 (21), 115 (16).

*Anti*-cyclooctadiene derivative **15**: ^1^H NMR (600 MHz, CDCl_3_) δ 6.45 (dd, 2H, *J*_1_ = 1.84, *J*_2_ = 7.80 Hz), 6.41 (d, 2H, *J* = 1.84 Hz), 6.31 (d, 2H, *J* = 7.80 Hz), 6.05–6.00 (m, 2H), 5.94–5.88 (m, 2H), 4.89–4.82 (m, 2H), 3.39–3.29 (m, 2H), 3.19–3.09 (m, 2H), 3.06–2.96 (m, 2H), 2.92–2.83 (m, 2H), 2.78–2.68 (m, 2H), 2.34–2.26 (m, 2H) ppm; ^13^C NMR (150.9 MHz, CDCl_3_) δ 143.6, 140.2, 139.4, 136.6 (+), 132.9 (+), 131.4 (+), 130.7 (+), 128.8 (+), 48.5 (+), 36.3 (−), 33.3 (−), 27.2 (−) ppm; MS (EI, 70 eV) *m*/*z* (%): 312 (19), 157 (33), 156 (40), 155 (100), 142 (12), 141 (55), 129 (18), 128 (22), 115 (17).

*Syn*-cyclooctadiene derivative **13**: ^1^H NMR (200 MHz, CDCl_3_) δ 6.40 (dd, 2H, *J*_1_= 1.68, *J*_2_ = 7.92 Hz), 6.30 (d, 2H, *J* = 1.68 Hz), 6.19 (d, 2H, *J* = 7.92 Hz), 5.91–5.72 (m, 4H), 4.57–4.40 (m, 2H), 3.39–3.22 (m, 2H), 3.19–2.83 (m, 4H), 2.82–2.49 (m, 4H), 2.33–2.11 (m, 2H) ppm; ^13^C NMR (50.3 MHz, CDCl_3_) δ 144.6, 139.7, 139.6, 139.4 (+), 134.4 (+), 130.8 (+), 130.4 (+), 129.5 (+), 54.2 (+), 36.4 (−), 33.6 (−), 27.7 (−) ppm; MS (EI, 70 eV) *m*/*z* (%): 312 (20), 157 (31), 156 (42), 155 (100), 142 (11), 141 (58), 129 (16), 128 (25), 115 (14).

**4,15-Bis[(*Z*)-2-cyclopropylvinyl][2.2]paracyclophane** (**22**): A freshly prepared solution of potassium *tert*-butoxide (898 mg, 8.0 mmol) in anhydrous THF (30 mL) was added dropwise over 30 min to a cooled (ice/water bath), vigorously stirred dispersion of cyclopropylmethyltriphenylphosphonium bromide (3178 mg, 8.0 mmol) in 30 mL of anhydrous THF under a N_2_ gas flow. The bath was removed and the mixture stirred for 1 h at ambient temperature, then re-cooled to 0 °C, after which a solution of **9** (264 mg, 1.0 mmol) in anhydrous THF (30 mL) was added dropwise over 1 h. The bath was removed and the mixture stirred for an additional 2 h. The resulting mixture was poured into a vigorously stirred mixture of ice (100 g), water (50 mL) and conc. (37%) aq. HCl solution (50 mL) and the mixture stirred until the ice had completely melted. The precipitate was suction filtered on a glass frit, washed with dilute (1:3) aq. HCl (3 × 30 mL) and water (3 × 30 mL), and dissolved in CH_2_Cl_2_ (50 mL): The organic solution was dried over Na_2_SO_4_, filtered and concentrated under reduced pressure without warming to give a colorless solid residue (333 mg, 98%) of hydrocarbon **22**, as a mixture of stereoisomers, pure by NMR. ^1^H NMR (200 MHz, CDCl_3_) δ 6.53–6.26 (m, 8H), 5.35 (dd, 0.04H, *J_1_* = 8.6, *J_2_* = 15.5 Hz), 5.24 (dd, 0.30H, *J*_1_ = 8.7, *J*_2_ = 15.6 Hz), 4.77 (dd, 1.36H, *J*_1_ = 10.3, *J*_2_ = 11.3 Hz), 4.75 (dd, 0.30H, *J*_1_ = 10.3, *J*_2_ = 11.5 Hz), 3.59–3.32 (m, 2H), 3.07–2.69 (m, 6H), 1.59–1.33 (m, 2H), 0.88–0.17 (m, 8H) ppm; ^13^C NMR for main isomer (50.3 MHz, CDCl_3_) δ 138.0, 137.4, 136.6, 135.0 (+), 133.6 (+), 132.9 (+), 130.9 (+), 126.5 (+), 34.4 (−), 32.7 (−), 10.4 (+), 6.8 (−), 6.5 (−) ppm; MS (EI, 70 eV) *m*/*z* (%): 340 (19), 171 (21), 170 (23), 169 (100), 155 (38), 142 (23), 141 (32), 129 (62), 128 (26), 115 (11).

Irradiation of 4,15-Bis[(*Z*)-2-cyclopropylvinyl][2.2]paracyclophane – [2.2.2]tricyclophanes **23** and **24**: A solution of **22** (51.0 mg, 150 µmol) was irradiated by a halogen torch lamp from a distance of 15 cm for 12 h. When the starting material had been completely consumed (TLC monitoring), the reaction mixture was separated by column chromatography (silica gel, pentane) to give 13.4 mg of *cis*-[2.2.2]tricyclophane **23** and 22.3 mg of *trans*-[2.2.2]tricyclophane **24**; total yield: 35.7 mg (70%).

*Cis*-[2.2.2]tricyclophane derivative (**23**): ^1^H NMR (200 MHz, CDCl_3_) δ 7.08 (d, 2H, *J* = 1.8 Hz), 6.46 (dd, 2H, *J*_1_ = 1.8, *J*_2_ = 7.8 Hz), 6.21 (d, 2H, *J* = 7.8 Hz), 4.53–4.41 (m, 2H), 3.21–2.89 (m, 6H), 2.69–2.29 (m, 2H), 1.66–1.48 (m, 2H), 0.79–0.64 (m, 2H), 0.59–0.46 (m, 2H), 0.24–0.09 (m, 4H) ppm; ^13^C NMR (50.3 MHz, CDCl_3_) δ 141.5, 140.0, 139.4, 136.4 (+), 133.1 (+), 127.9 (+), 48.4 (+), 46.8 (+), 36.5 (−), 33.3 (−), 9.8 (+), 7.8 (−), 4.7 (−) ppm; MS (EI, 70 eV) *m*/*z* (%): 340 (15), 171 (22), 170 (24), 169 (100), 155 (33), 142 (20), 141 (27), 129 (51), 128 (22), 115 (11).

*Trans*-[2.2.2]tricyclophane derivative (**24**): ^1^H NMR (200 MHz, CDCl_3_) δ 6.44 (dd, 2H, *J*_1_ = 1.7, *J*_2_ = 7.8 Hz), 6.19 (d, 2H, *J* = 7.8 Hz), 6.15 (d, 2H, *J* = 1.7 Hz), 4.16–4.00 (m, 2H), 3.26–2.89 (m, 4H), 2.60–2.45 (m, 2H), 2.26–2.10 (m, 2H), 1.48–1.31 (m, 2H), 0.74–0.41 (m, 4H), 0.30–0.07 (m, 4H) ppm; ^13^C NMR (50.3 MHz, CDCl_3_) δ 141.4, 140.5, 140.2, 136.4 (+), 133.6 (+), 129.0 (+), 50.3 (+), 41.7 (+), 37.0 (−), 33.2 (−), 13.5 (+), 5.2 (−), 4.1 (−) ppm; MS (EI, 70 eV) *m*/*z* (%): 340 (15), 171 (21), 170 (21), 169 (100), 155 (27), 142 (11), 141(19), 129 (44), 128 (20), 115 (10).

### X-ray structure determination

Numerical details are presented in Table 1. Data collection and reduction: Crystals were mounted in inert oil on glass fibres and transferred to the cold gas stream of the appropriate Oxford diffractometer. Measurements were performed with monochromatic Mo-*K*α (λ = 0.71073 Å; **23**) or mirror-focussed Cu-*K*α radiation (λ = 1.54184 Å; all others). Absorption corrections were performed for the Cu data sets only, on the basis of multi-scans. Structure refinement: The structures were refined anisotropically against *F*^2^ (program SHELXL-97 [25]). Hydrogen atoms were included with a riding model. *Exceptions and special features*: For **23**, hydrogen atoms of the three- and four-membered rings were refined freely but with C–H distance restraints. For (*Z*,*Z*)-**22** and **23**, which crystallize in non-centrosymmetric space groups, anomalous scattering was negligible and Friedel opposite reflections were therefore merged. For **24**, the atoms C23–26 show a slight (9%) disorder. The disorder model was refined using a system of similarity restraints. Dimensions of the minor disorder component should be interpreted with great caution.

**Table 1:** Crystallographic data for compounds **13**, **15**, (*Z*,*Z*)-**22**, **23** and **24**.

Compound	**13**	**15**	(*Z*,*Z*)**-22**	**23**	**24**

Formula	C_24_H_24_	C_24_H_24_	C_26_H_28_	C_26_H_28_	C_26_H_28_
*M*_r_	312.43	312.43	340.48	340.48	340.48
Habit	colourless prism	colourless plate	colourless tablet	colourless tablet	colourless lath
Cryst. size (mm)	0.2 × 0.1 × 0.08	0.08 × 0.06 × 0.015	0.25 × 0.2 × 0.1	0.4 × 0.35 × 0.2	0.25 × 0.04 × 0.01
Crystal system	monoclinic	monoclinic	orthorhombic	monoclinic	monoclinic
Space group	*P*2_1_/*c*	*P*2_1_/*c*	*P*2_1_2_1_2_1_	*C*2	*P*2_1_/*c*
Cell constants:					
*a* (Å)	11.4023(3)	17.4745(12)	7.75839(15)	20.1622(5)	12.4933(5)
*b* (Å)	7.5646(2)	8.4668(6)	15.1450(2)	8.1838(2)	7.5363(3)
*c* (Å)	19.2444(5)	11.3625(7)	32.3931(6)	12.4063(3)	19.7420(7)
α (°)	90	90	90	90	90
β (°)	92.696(3)	104.052(7)	90	117.394(4)	96.997(4)
γ (°)	90	90	90	90	90
*V* (Å^3^)	1658.08	1630.8	3806.23	1817.54	1844.93
*Z*	4	4	8	4	4
*D*_x_ (Mg m^−3^)	1.252	1.273	1.188	1.244	1.226
μ (mm^−1^)	0.52	0.53	0.50	0.07	0.51
*F*(000)	672	672	1472	736	736
*T* (°C)	−173	−173	−173	−173	−173
Wavelength (Å)	1.54184	1.54184	1.54184	0.71073	1.54184
2θ_max_	152	146	152	61	152
Refl. measured	34233	25579	69531	60932	35396
Refl. indep.	3449	3218	4469	2945	3839
*R*_int_	0.029	0.087	0.025	0.031	0.056
Parameters	217	217	469	291	252
Restraints	0	0	0	44	29
*wR*(*F*^2^, all refl.)	0.105	0.109	0.088	0.090	0.126
*R*(*F*, >4σ(*F*))	0.041	0.043	0.033	0.032	0.045
*S*	1.07	0.97	1.04	1.05	1.07
max. Δ/ρ (e Å^−3^)	0.35	0.26	0.20	0.31	0.28

Crystallographic data have been deposited with the Cambridge Crystallographic Data Centre as supplementary publications no. CCDC-797335 (**13**), -797336 (**15**), -797337 (*Z*,*Z*-**22**), -797338 (**23**), -797339 (**24**). Copies of the data can be obtained free of charge from http://www.ccdc.cam.ac.uk/data_request/cif.
